# Biosafety of GM Crop Plants Expressing dsRNA: Data Requirements and EU Regulatory Considerations

**DOI:** 10.3389/fpls.2020.00940

**Published:** 2020-06-24

**Authors:** Salvatore Arpaia, Olivier Christiaens, Kara Giddings, Huw Jones, Bruno Mezzetti, Felix Moronta-Barrios, Joe N. Perry, Jeremy B. Sweet, Clauvis N. T. Taning, Guy Smagghe, Antje Dietz-Pfeilstetter

**Affiliations:** ^1^ENEA, Italian National Agency for New Technologies, Energy and Sustainable Economic Development, Rotondella, Italy; ^2^Department of Plants and Crops, Faculty of Bioscience Engineering, Ghent University, Ghent, Belgium; ^3^Bayer, Crop Science R&D Regulatory Science, Chesterfield, MO, United States; ^4^Translational Genomics for Plant Breeding, Aberystwyth University, Wales, United Kingdom; ^5^Department of Agricultural, Food and Environmental Sciences, Università Politecnica delle Marche, Ancona, Italy; ^6^Regulatory Sciences, ICGEB, Trieste, Italy; ^7^Oaklands Barn, Norfolk, United Kingdom; ^8^JT Environmental Consultants Ltd, Cambridge, United Kingdom; ^9^Institute for Biosafety in Plant Biotechnology, Julius Kühn-Institut, Federal Research Centre for Cultivated Plants, Braunschweig, Germany

**Keywords:** RNA interference, biosafety, food safety, genetically modified plants, bioinformatics, non-target organisms, GMO regulation

## Abstract

The use of RNA interference (RNAi) enables the silencing of target genes in plants or plant-dwelling organisms, through the production of double stranded RNA (dsRNA) resulting in altered plant characteristics. Expression of properly synthesized dsRNAs in plants can lead to improved crop quality characteristics or exploit new mechanisms with activity against plant pests and pathogens. Genetically modified (GM) crops exhibiting resistance to viruses or insects *via* expression of dsRNA have received authorization for cultivation outside Europe. Some products derived from RNAi plants have received a favourable opinion from the European Food Safety Authority (EFSA) for import and processing in the European Union (EU). The authorization process in the EU requires applicants to produce a risk assessment considering food/feed and environmental safety aspects of living organisms or their derived food and feed products. The present paper discusses the main aspects of the safety assessment (comparative assessment, molecular characterization, toxicological assessment, nutritional assessment, gene transfer, interaction with target and non-target organisms) for GM plants expressing dsRNA, according to the guidelines of EFSA. Food/feed safety assessment of products from RNAi plants is expected to be simplified, in the light of the consideration that no novel proteins are produced. Therefore, some of the data requirements for risk assessment do not apply to these cases, and the comparative compositional analysis becomes the main source of evidence for food/feed safety of RNAi plants. During environmental risk assessment, the analysis of dsRNA expression levels of the GM trait, and the data concerning the observable effects on non-target organisms (NTO) will provide the necessary evidence for ensuring safety of species exposed to RNAi plants. Bioinformatics may provide support to risk assessment by selecting target gene sequences with low similarity to the genome of NTOs possibly exposed to dsRNA. The analysis of these topics in risk assessment indicates that the science-based regulatory process in Europe is considered to be applicable to GM RNAi plants, therefore the evaluation of their safety can be effectively conducted without further modifications. Outcomes from the present paper offer suggestions for consideration in future updates of the EFSA Guidance documents on risk assessment of GM organisms.

## Introduction

In the last decade, a variety of new biotech methods have been developed, offering great technical potential for use in the agricultural sector. The European Union has established a legal framework to ensure that the application of modern biotechnology, and more specifically of genetically modified organisms (GMO), is developed under safe conditions. According to the EU legislation on GMO, in particular Regulation (EC) No 1829/2003 on genetically modified (GM) food & feed and Directive 2001/18/EC on deliberate release into the environment of GMO, every application for commercial use of a GMO has to be approved by the European Commission after a safety assessment. This assessment is conducted by the applicants according to the requirements of either of the two laws. For the preparation of dossiers, applicants need to conduct appropriate experimental studies and literature searches for collecting information regarding human and animal health, as well as environmental safety. The data requirements according to EU legislation on GMOs are laid down in the implementing Regulation No 503/2013 and Commission Directive (EU) 2018/350 for food/feed and environmental safety respectively. Support for applicants in preparing dossier for the commercialization of GM products is guaranteed by specific guidance documents (GDs) issued by the European Food Safety Authority (EFSA Panel on GMO, 2010; EFSA Panel on GMO, 2011) and periodically updated or supplemented by specific statements following the latest scientific developments in the field. As new biotech products are being developed, it is important that the rationale for the safety assessment is built on a scientific consideration of the characteristics of the new products in accordance with the principles of the EFSA GDs.

The RNA-interference (RNAi) technique exploits a natural mechanism present in almost all eukaryotic organisms, which leads to the loss of functionality of a gene by blocking the messenger RNA (mRNA) molecules essential for the formation of a protein. The RNA expression constructs are normally delivered as transgenes, *via* plant transformation or as a part of virus‐vectors (Yin et al., 2005), and therefore, they are required to undergo GMO regulatory procedures to enable authorization for commercial use. However, unlike classical GM plants that are generally modified to express a specific protein, GM plants expressing dsRNA (hereafter, RNAi plants) do not need to express novel proteins to produce a new phenotype. RNAi plants have been modified to express double stranded RNA (dsRNA) molecules that enable specific post-transcriptional partial or complete silencing of plant target genes or target genes from plant pathogens or pests.

RNAi can be used in a “within species” mode to improve plant composition by removing or reducing anti-nutrients, allergens and toxins while enhancing levels of beneficial nutrients, and to improve plant growth and productivity by suppressing undesirable traits. The same mechanism can be exploited by expressing dsRNA in plants that silence genes in other organisms exposed to the plants (Zotti et al., 2018). Virus resistant and insect resistant plant varieties obtained using RNAi mechanisms have already been approved for cultivation outside Europe (e.g. papaya resistant to Papaya Ringspot Virus in USA, Canada and Japan; plum tree resistant to plum pox virus (PPV) approved in the USA; common bean resistant to Bean Golden Mosaic Virus (BGMV) in Brazil; SmartStax™ maize with multiple resistance traits, including dsRNA against *Diabrotica virgifera virgifera* approved in the USA and Canada), cassava resistant to Brown Streak Virus and Ugandan Cassava Brown Stick Virus in Nigeria. In the EU, several RNAi plants with enhanced nutritional characteristics including Soybean DP305423 and soybean MON87705 with increased oleic acid acid have been authorised for placing on the market with the exception of cultivation. More recently, EFSA has given a positive opinion for import and processing of products derived from corn rootworm resistant GM maize MON87411 (EFSA Panel on GMOs, 2018). No applications for cultivation of plants producing dsRNA have been submitted so far to European authorities. However, EFSA has published an opinion on a GM potato event with antisense-mediated gene silencing (EFSA, 2006).

As knowledge in this field is rapidly accumulating, EFSA has supported the publication of 3 systematic literature searches and reviews, summarizing the available information on RNAi technology to support the risk assessment of RNAi plants (Paces et al., 2017; Christiaens et al., 2018; Dàvalos et al., 2019). While these studies did not directly offer any recommendations for risk assessment, they offer valuable baseline information supporting any future risk assessment framework. Previously, EFSA had also organized an international workshop on “Risk assessment considerations for RNAi-based GM plants” (European Food Safety Authority, 2014) which also led to a number of external peer-reviewed publications discussing the risk assessment considerations of these plants (Ramon et al., 2014; Casacuberta et al., 2015). Together also with EPA’s FIFRA Scientific Advisory Panel meeting publication (USEPA, 2014), these publications form the first wave of risk assessment considerations.

In the field of pest management, RNAi applications are exploring completely novel mechanisms of actions, as has been shown from work on RNAi plants as well as from direct applications of dsRNA as pesticide product. Effective examples are: silencing of the *DvSnf7* gene, which results in suppression of mRNA encoding the class E vacuolar sorting protein in *D. v. virgifera* (Bachman et al., 2013); targeting of vacuolar-ATPase subunit A (v‐ATPase A) in *D. v. virgifera* (Baum et al., 2007) and in *Bemisia tabaci* (Thakur et al., 2014) by the expression of dsRNA. Furthermore, examples of RNAi-based control to manage filamentous fungal plant pathogens are steadily increasing (Machado et al., 2018). Progress in the development of RNAi plants and also new RNA-based biopesticides is expected to bring to the market new plant varieties or plant protection products within a few years (Taning et al., 2020).

The COST Action iPlanta (iplanta.univpm.it) is the largest network of European scientists actively engaged in research on RNAi systems and applications, including host-induced gene silencing (HIGS) and spray induced gene silencing (SIGS). Among the goals of iPlanta are the identification of the specific biosafety data requirements for the risk assessment and risk management of RNAi plants and their products (i.e. food and feed) and the elucidation of knowledge gaps arising in the area of potential food and feed and/or environmental risks specific to RNAi applications.

The present paper is the result of an iPlanta working group activity and aims to discuss the relevance and applicability of the existing EFSA GMO Guidelines for environmental and food/feed risk assessment for RNAi plants. Starting from some key elements of the EFSA GDs, the paper discusses the applicability of the principles of the GDs to RNAi plants and, based on the considerations of their scientific aspects involved, suggests the data requirements considered significant for preparation of dossiers.

## Comparative Assessment

In the EFSA GDs, comparative safety assessment is indicated as a general principle for the risk assessment of GM plants. The GDs therefore suggest optimal methodologies for data collection on relevant assessment endpoints, to compare GM plants and derived food and feed, with their respective comparators (e.g. the near isogenic control line). The comparative safety assessment of GM plants is considered effective to identify differences to their non-GM counterparts and assess the consequences of these differences.

Comparative safety assessment is based on data collected for molecular characterization, the agronomic and phenotypic characteristics of the GM plant, as well as its compositional analysis. In addition, the comparative safety assessment within environmental risk assessment (ERA) requires information on the interactions of the GM plant with other biota and its receiving environment(s).

The main requirements and questions raised by the EFSA GD are the following:

- Describe how the tested GM line was produced (breeding tree);
- Document that the selected conventional counterpart is genetically as close as possible to the GM line and has a history of safe use;
- Select (a minimum of 8) experimental sites for conducting field experiments concerning compositional, agronomic and phenotypic analysis, where the meteorological and agronomic conditions reflect the ones under which the crop is to be grown;
- Select appropriate plant genotypes, e.g. the GM line genetic background to ensure the quality and stability of the selected test material;
- Include in the field experiments the GM line(s), the near isogenic control and 3 reference commercial varieties at each site (minimum 6 different varieties in total), on the basis of having adequate statistical power to detect differences or equivalence;
- Indicate the measurement endpoints to be used;

These power calculations depend respectively on the typical variability found between small agronomic plots used for food-feed trials and between the larger plots typically employed for environmental trials (see EFSA Panel on Genetically Modified Organisms, 2010; EFSA Panel on Genetically Modified Organisms, 2011).

In neither case is the methodology of the field trial assessment related to the technology adopted to produce the novel plant or food under assessment. For the above-mentioned points, it is considered that the implementation of field experiments for RNAi plants will not be different from all currently grown GM events.

It could be problematic to field test RNAi plants with tolerance to extremes of environmental conditions such as temperature, salinity and soil moisture, but this is also the case for other GM plants. In addition, comparative field testing in the presence of a target pathogen or pest may not be easily realized. For instance, in the case of virus resistant RNAi plants interactions between GM plants and the targeted viruses have to be assessed in preliminary tests. It is also important to consider that the composition of an RNAi plant may be altered after the attack by a specific pest or pathogen due to general and dsRNA- specific defence responses, which may change plant physiology/metabolism (Eschen-Lippold et al., 2012). Consequently, specially designed field studies located in regions with high pathogen or pest pressure are useful to assess the safety of newly introduced plant or pest resistance mechanisms under typical cultivation conditions and to detect any possible unintended effects.

The current EFSA GD have been used mostly to consider applications for herbaceous GM plants. However perennial RNAi plants such as virus resistant RNAi GM trees (e.g. Rainbow papaya, Ferreira et al., 2002, Honey sweet plum, Scorza et al., 2013), may require other measurement endpoints relating to their development and seasonal growth in comparative field studies for safety evaluations (Aguilera et al., 2013).

In some applications (e.g. virus resistance in GM trees) dsRNA can be used for transforming rootstocks so that the effects occur in the whole plant, because of the translocation of siRNA into scions grafted to the transformed rootstocks (Limera et al., 2017; De Francesco et al., 2020). Several studies demonstrated the transfer of siRNAs from the transgenic rootstock to the nontransgenic scion conferring resistance to virus in fruit trees (Zhao and Song, 2014) and grafted cucumber (Bai et al., 2016). This technology is now studied also for conferring resistance to pests (Taning et al., 2020) and diseases (Sabbadini et al., 2019). Such systemic silencing probably depends on amplification of siRNAs through secondary siRNA production. However, in contrast to highly expressed or aberrant RNAs (such as viral RNAs or RNA from transgenes), endogenous plant mRNAs are usually not prone to secondary siRNA production (Luo and Chen, 2007; Baeg et al., 2017) and to systemic silencing (Frizzi and Huang, 2010; Dadami et al., 2014). Therefore, translocation of siRNAs to scions and fruits and the usage of grafting technologies will be available only for specific applications.

## Molecular Characterization

According to the strategy of the EFSA GDs, following a case-specific problem formulation, the risk assessment starts with the comprehensive molecular characterization of the GM plant under scrutiny.

The main areas on which information is required during the molecular characterization are:

- Information on the intended genetic modification which includes: the transformation process (method of transformation; recipient plant tissue; details of *Agrobacterium* strain used, helper plasmids etc.); the source of DNA and design of vector constructs (sequence of DNA to be inserted, codon optimisation, promoters etc.); the role of each functional element
- Information on the GM plant, including: the trait(s) modified; the transgene constructs actually present in the GM plant; protein characterization and equivalence; expression and stability of the insert.
- Bioinformatics data/open reading frame analysis to identify potential for newly created toxins/allergens.

These data also inform the risk analysis of horizontal gene transfer (see below).

Data concerning the transformation process and the sequence and function of the inserted DNA for RNAi plants are not different from other transgenic events. Also, where the RNAi event possesses additional conventional transgenes for selection etc., the normal data requirements still apply to those parts.

However, for the inserted dsRNA cassette specifically, some of the data normally required for GM plants simply cannot be obtained. For instance, data related to newly expressed proteins, protein equivalence and the codon optimization are irrelevant for this inserted DNA as long as no part is translated into protein. Data on levels of dsRNA expression over time and plant development stages in different tissues and environmental conditions relevant for the crop in question will be necessary to estimate exposure of humans and other animals to the dsRNA in food or feed. dsRNA levels in plant tissues are also dependent on the type of transformation. In nuclear transformants dsRNA is to a large part processed in plant cells into siRNAs (Frizzi and Huang, 2010) which are not efficiently taken up by insects. In contrast, transplastomic plants accumulate dsRNA within the chloroplasts (Bally et al., 2016), resulting in high amounts of unspliced dsRNA in green plant parts (Zhang et al., 2015) and may therefore be used particularly for targeting leaf-feeding insect pests. Exposure of organisms to dsRNA through roots, tubers and pollen, however, is low in transplastomic RNAi plants, which implies that nuclear transformants and transplastomic plants are likely to have different risks for non-target organisms (Schiemann et al., 2019). Bioinformatics analyses offer the availability of additional measurement endpoints, which can be potentially more specific for events transformed with dsRNA. According to EU implementing regulation 503/2013, bioinformatics analyses are requested for the recipient plant genome to detect potential off-target plant genes that might be suppressed unintentionally. Considerations including a set of parameters that allow the prediction of possible off-target transcripts in plants have been published by the EFSA GMO Panel (EFSA GMO Panel (2017) Annex II of the minutes of the 118th GMO Plenary meeting: Internal note on the strategy and technical aspects for small RNA plant off-target bioinformatics studies. Available at: https://www.efsa.europa.eu/sites/default/files/event/171025-m.pdf). On a case-by-case basis and depending on the function of the potential off-targets, additional data may be required for safety assessment.

Such information could also be considered, although with a more limited value, for estimating possible off-target effects in outcrossing plant species or non-target organisms in the receiving environment, however this is feasible only when relevant genomic information is available on organisms exposed to the GM plant or its dsRNA.

A key benefit of the sequence-based mechanism of action for RNAi plants for pest control is the possibility to achieve a high degree of specificity to the target organism while not harming exposed valued NTOs in the agroecosystem (Bramlett et al., 2020). Bioinformatics are informative for the selection of regions within target genes that possess high divergence across species, thus allowing for the selection of gene sequences specific to the target pest and minimizing the potential for homology to NTOs that are potentially exposed to the GM plants or its products. In order to achieve this, the search for 21bp homologies between the dsRNA and possible target sequences in NTOs is a good starting point, but the exact length and sequence of the construct that may produce gene suppression in different invertebrate species are not precisely known. Paces et al. (2017) indicates that while siRNA-target base pairing is highly specific, mismatches do not necessarily prevent RNAi silencing, depending on position and type of mismatch. Furthermore, available literature is in disagreement regarding the minimum effective base pairs length, number and location of allowed mismatches (Christiaens et al., 2018). SiRNAs also appear to vary in length in different insect species. A recent research paper looking at siRNA populations after viral infections showed two tested lepidopteran species having predominantly 20nt siRNAs while siRNAs in the examined orthopteran and hymenopteran species were mostly 22nt long (Santos et al., 2019). This could imply that the necessary homology for successful RNAi silencing could differ between species as well. Coleoptera typically have 21nt long siRNAs and research experience supporting the development of the MON87411 maize with dsRNA targeting the *DvSnf7* gene, indicates that shorter than 21 nt sequence length shared between the dsRNA construct and the target gene (19, 20 bp) did not result in an efficient silencing effect in the target insect (Bachman et al., 2013).

Finally, it must be noted that little sequence information is available for many NTOs and factors other than sequence homology (e.g. successful uptake and stability of the dsRNA molecules into the insect, accessibility of the mRNA site of action) may affect the efficacy of siRNA. Therefore, it may be necessary to generate data on the effects of the dsRNA on exposed NTOs during ERA. This is discussed below.

## Food/Feed Safety Assessment

The main pillars on which is based the safety assessment of food/feed containing or derived from GMOs according to the EFSA GD (EFSA Panel on GMO, 2011) are:

- Toxicological assessment of newly expressed proteins and/or new constituents other than proteins;
- Assessment of allergenicity of the newly expressed proteins and the new plant;
- Safety assessment of altered levels of food and feed constituents;
- Safety assessment of the whole food and/or feed derived from GM plants;
- Nutritional assessment of food/feed derived from GM plants.

No new proteins are intended to be produced by the dsRNA cassette in RNAi plants, therefore the toxicological assessment of newly expressed proteins is not relevant for food/feed products derived from them. Consequently, since all known food allergens are proteins, the allergenicity can also generally be considered not a relevant concern for RNAi plants, unless expression of genes coding for enzymes involved in the metabolism of existing plant allergens are silenced in the GM plant.

Compositional analyses can then constitute the key requirement for analysing the effects of identified differences, as well as for nutritional evaluation of food and feed derived from RNAi plants. Indeed, major metabolic changes in comparison with the near-isogenic control plants can be detected with compositional analyses, for which international standards are available and commonly applied for food safety assessment. For instance, the Organisation for Economic Co-operation and Development (OECD) consensus documents on the safety assessment of transgenic organisms (http://www.oecd.org/env/ehs/biotrack/safetyassessmentoftransgenicorganismsoecdconsensusdocuments.htm, accessed on 12 December 2019) include proximates (comprising moisture and total ash), key macro- and micro-nutrients, anti-nutritional compounds, natural toxins, and allergens, as well as other plant metabolites characteristic for the plant species.

For the safety assessment of whole food/feed, animal feeding trials are recommended by EFSA only on a case-by-case basis, specifically those cases for which the quality of available analytical data does not allow excluding possible safety issues for the specific product or fail to demonstrate nutritional equivalence with its comparator. While 90-days feeding studies are deemed effective methods to detect toxic effects of single substances, there has been considerable discussion over their relevance and sensitivity for the detection of potential unintended effects of whole food and feed. It is considered unlikely that substances present in small amounts and with a low toxic potential will result in any observable unintended effects in a 90-day rodent feeding study (EFSA, 2008). However, following the adoption of the Implementing Regulation (EU) 503/2013, a 90-day study in rodents on whole food/feed is required for all GM plant products in the EU. This new legal requirement changes the assessment from a hypothesis-driven case-by-case exercise, as originally indicated by the EFSA GD.

Exposure to dsRNA and siRNA through GM RNAi plant-derived food/feed by humans and farm animals is estimated to be low, due to some considerations regarding the metabolism of organisms ingesting exogenous RNA (Dàvalos et al., 2019). First of all, the uptake of ingested exogenous nucleic acids is limited by biological barriers in the gastro-intestinal (GI) tract, such as degradation by nucleases or an impaired cellular uptake (e.g. O’Neill et al., 2011; Petrick et al., 2013). Moreover, RNA absorption from the GI tract remains questionable (Jain, 2008; Thompson et al., 2012). Even if ingested siRNA is absorbed from the GI tract, it is normally rapidly degraded within the cardiovascular system and cleared through liver and kidneys (Christensen et al., 2013). These barriers represent the main difficulty for the development of targeted human RNAi drugs (e.g. Vaishnaw et al., 2010) which require specific formulations to deliver siRNA into the target cells. Consequently, it is unlikely that siRNA concentrations from GM RNAi plants will be sufficient to exert biologically relevant effects in mammals. In order to lead to harmful effects, the uptake of dsRNA should be followed by the delivery in sufficient quantity and in an active form to trigger RNAi and there would also need to be sufficient sequence complementarity with an mRNA transcript in the targeted cells (Roberts et al., 2015). Therefore, the risk of unwanted gene silencing in humans and animals upon ingestion of food/feed derived from RNAi plants can be considered negligible.

## Environmental Safety Assessment

The EFSA GD on Environmental Risk Assessment (EFSA Panel on GMO, 2010) requires a safety evaluation regarding different specific areas of concern.

- **Persistence and Invasiveness**
  Cultivated plants may persist in the environment even after harvest and some crop species have wild relatives with which they can hybridize allowing genes to flow from crops into other species. However, gene flow frequency and its consequences are very variable. For instance, gene flow is already an important issue in open pollinating plants with common wild relatives e.g., rice and beet but not in predominantly self-pollinating species such as wheat or beans. The focus of the ERA is on the expected consequences of gene flow, once it happens.
  The information needed for assessing consequences of plant transformation on persistence and invasiveness is based on the following points in order to analyse the possibility of environmental harm:
  - Understand the biological features of the plant that has been genetically modified (e.g. life cycle, dispersal, gene flow, persistence, invasiveness, etc.);
  - Analyse how the transgene affects the phenotype, behaviour, and interaction of the GM plant with the hybridizing wild relatives present in the receiving environment.
  The likelihood of increased persistence of the RNAi plant and recipient wild relatives in the environment is linked to the expressed traits of the GM plant, therefore the assessment for RNAi plants is similar to that of other GM plants and is case by case.
  The possibility of off-target effects in wild relatives of the GM plant could be estimated starting from the provided bioinformatics results and the acquired characteristics of the transformed plant species. Bioinformatics could be helpful as a predictive tool to detect possible off-target sequence alignment considering the similarities between the genome of the GM donor crop and the recipient plant. However, information on the genome of the wild relatives might be only partially available or completely lacking in the scientific literature.
  If transgene transfer to a wild relative occurs, then NTOs associated with the wild plant may be exposed to the dsRNA and should be considered in the safety assessment. The expression and environmental persistence of dsRNA in different tissues over time needs attention, as their environmental stability is expected to be different (Christiaens et al., 2018). However, this exposure pathway is not specific to RNAi plants as similar data may be required for GM plants expressing other biologically active compounds such as Cry proteins.

- **Horizontal Gene Transfer**
  The main issues to be considered in this case are:
  - Molecular characterization of the DNA sequences inserted in the plant, including information on the potential of the promoter elements that could drive expression in microorganisms;
  - presence of antibiotic resistance marker (ARM) genes;
  - presence of recipient microorganisms for transgenic DNA in the receiving environments;
  - presence of inserted DNA sequences showing homologies with DNA sequences from relevant microbial recipients, enhancing the probability of recombination, or mobile elements in the vicinity of the insertion site which could enhance the potential for gene transfer;
  - selective conditions enhancing the probability of dissemination and maintenance of the genetic material from GM plants in natural microbial communities (e. g. the presence of antibiotics in the receiving environment(s);
  - environmental persistence of GM plant material after harvesting;
  - potential for long-term establishment of the genetic materials from GM plants in microbial communities;
  - ecological or human/animal health consequences of a potential HGT from a GM plant to microorganisms (e.g. potential spread of antibiotic resistance genes and probability of reduced efficiency of antibiotic treatments in humans);
  - information on the prevalence and distribution of genes identical or similar to the transgene in microorganisms in natural environments.
  The likelihood of DNA transfer to bacteria is generally independent of the function of the DNA sequence, therefore the probability is the same as for other transgenes. However, integration of transferred DNA into the bacterial genome by homologous recombination depends on sequence homologies. For sequences that encode dsRNA targeting genes of plants or plant pests the likelihood for sufficient sequence homologies to a bacterial genome is rather low. As for other GM plants, if regulatory sequences from bacteria are present in the transgene construct (like the nopaline synthase promoter and terminator from *Agrobacterium tumefaciens*), they may provide homologies necessary for integration into the genome of microorganisms. Maintenance of transferred DNA in bacteria is dependent on the encoded trait and on possible selective conditions (i.e. transfer of an antibiotic resistance marker gene can allow a selective advantage in the presence of the respective antibiotic). One of the differences between RNAi plants and a conventional GM plant is that the newly inserted DNA does not code for a protein. This implies that in case of horizontal DNA transfer from RNAi plants to bacteria no new functions will be acquired through expression of a novel protein. In addition, bacteria do not possess the RNAi machinery that is homologous to eukaryote cells so targeted modification of gene expression is not easily possible.
  Gene transfer is a rare event, but frequency may increase if there is positive selection of the transferred sequences to levels where it may be relevant. Potential ecological consequences due to the transfer to microorganism of a gene or trait that is already widespread in the environment (like many of the ARM genes) need to be estimated case by case; however, also this aspect is not specifically related to RNAi plants.
  For some of the GM events transformed with dsRNA currently authorized in non-EU countries (e.g Rainbow papaya 55-1, Arctic™ apple), antibiotic resistance marker genes are included in the cassette (http://www.isaaa.org/gmapprovaldatabase/default.asp); however, there are no reports concerning negative effects on the spread of ARM genes in the environments where these crops are being cultivated. Other RNAi events were obtained without insertion of ARM genes in the cassette. This prevents concerns for possible spreading of ARM genes and can be confirmed with molecular characterization of every newly developed event.
  Exposure characterization steps are similar for all GM plants, no specific additional requirements are deemed necessary and the existing requirements are considered equally applicable.

- **Target and Non-Target Organisms**
  The main questions raised by the EFSA ERA GD are the following:
  **Target Organisms**
  - Data on the exposure of target organisms to the GM plant;
  - Data on the potential for resistance development in the target organisms.
  Exposure data describing dsRNA expression in different plant parts are derived from the compositional and molecular characterisation of GM RNAi plants as previously discussed. This will show possible exposure levels of targeted plant pests and pathogens throughout the growing season and post-harvest in seeds, plant biomass and plant debris. dsRNA may be designed to have sub-lethal effects on target species, for example by preventing mature development or inducing sterility. Thus *in vivo* studies involving *in planta* exposure are required to determine effects at both individual and population level.
  Continuous or repeated exposure exerts a selection pressure which can promote onset of resistance in target pest populations. RNAi mechanisms are not an exception to this general rule. Khajuria et al. (2018) reported the first known case of a pest resistant to a mechanism of gene silencing. The authors selected in laboratory conditions a strain of the western corn rootworm, *D. v. virgifera* resistant to dsRNA targeting *DvSnf7* gene, through exposure to GM maize MON 87411. The study demonstrated that the resistance mechanism is based on an altered uptake of RNA molecules and individuals of this colony showed cross-resistance also to other dsRNA tested in their experiments.
  Though the insurgence of resistance in target organisms represents mainly an agronomic problem, a possible drawback for the environment is the fact that the spread of a resistant strain could damage crop species in the area and then require additional use of pesticides. Data necessary for estimating the potential for resistance development in target organisms refer to the biology of target organisms (e.g. allele frequency, fitness, mobility). Data requirements for estimating the exposure of target organisms pertain to the ecology of the pest species and the levels of exposure to dsRNA in plant parts. In cases of RNAi plants where a dsRNA targets an insect pest gene and the plant also expresses a Cry toxin targeting the pest (e.g. as in maize MON 87411), the insects ability to develop resistance to the GM plant will be influenced. The resistance management strategies of EFSA (EFSA, 2015) apply here, and post release monitoring plans should therefore consider the likelihood of resistance development to such pyramided or stacked traits.
  *In planta* tests are applicable for the study of pathogen resistance induced *via* RNAi (Rosa et al., 2018), for instance, by modulating the expression of membrane surface proteins. This type of response has to be tested in actively growing plants as surface protein responses may vary with plant development, and this may have different impacts on the infectivity of pathogens.
  **Non-Target Organisms**
  The steps needed to conduct an environmental safety assessment according to the EFSA ERA GD are the following:
  - Collection of available knowledge of the environments and ecosystems likely to be exposed to the RNAi plants or plant parts or to hybridising relatives;
  - Selection of NTO focal species to be tested based on the presence and ecological relevance of the species occurring in receiving environments of the GM plant, their sensitivity to the potential stressor and likely to be exposed to the dsRNA either through the GM plant or food chains;
  - selection of measurement endpoints representative for protection goals;
  - setup experimental protocols for bioassays to assess direct and indirect effects on selected NTO;
  - data collection for estimating the exposure of NTOs to the dsRNA in GM plant and food chains.

The criteria for the selection of non-target focal species are based on the ecology of the NTOs, therefore no different assessment is needed compared to any other GM events, however bioinformatics can provide support to the selection procedure. Information on the presence of the same gene sequences targeted by the dsRNA in the genome of NTO can be used to select species potentially sensitive to the dsRNA expressed in GM plants. However, sequence match does not necessarily mean risk as the organism possess digestive and other physiological barriers that degrade/exclude the dsRNA from being taken up. Given the large variability in RNAi efficacy in invertebrates, even between closely related species sometimes, the species’ sensitivity to RNAi could also be taken into account during the selection procedure. Species known as being barely sensitive to dsRNA after oral uptake are less valuable as NTOs in risk assessment.

In order to estimate the exposure potential of NTOs, in addition to some pheno-ecological characteristics of the NTO and its host plants (e.g. host spatial distribution and life cycle, overlap of NTO and plant life cycle) it is necessary that the presence of dsRNA/siRNA in plant tissues over time is estimated. While the full-length dsRNA is expressed in plants and can be reliably measured across tissues, the endemic dicer in plants will result in pools of siRNAs. siRNA estimates may not be relevant, since they are not expressed, but become biologically available upon metabolic activation of the RNA machinery. Specifically, levels of siRNAs in plant tissues would likely be pools and therefore of limited biological impact, which can be better assessed with bioassays. Measuring the dsRNA instead, can provide a better estimate for exposure, based on the assumption that no greater number of siRNAs would be present than starting dsRNA. The important information is the minimum amounts of dsRNA required to initiate siRNA silencing activity, once that threshold is reached there may not be a direct correlation between quantities of dsRNA and activity levels. Therefore, quantification of full-length dsRNA provides the most appropriate exposure value for NTOs.

Organisms at the third trophic level (e.g. parasitoids, predators), can also be exposed indirectly to dsRNA by feeding on herbivore hosts/preys. The movement and the residual activity of dsRNA at the third trophic level was detected by Garbian et al. (2012) in *Varroa destructor* individuals feeding on *Apis mellifera*. It is known that some predator species show sensitivity to dietary RNAi upon ingestion (Haller et al., 2019), however the occurrence of such effects if exposed to a natural feeding regime (i.e. ingestion of prey feeding on dsRNA containing diet and pollen) in controlled conditions needs to be confirmed. To date the tritrophic bioassay with *Varroa* mites represents the only studied case in which exposure at the third trophic level was experimentally demonstrated and therefore specific bioassays will be needed to prove actual exposure of non-target carnivores.

Experimental protocols for evaluating effects on NTOs may need to be adapted to the case of RNAi plants. The most relevant route of exposure for NTOs in nature is likely to be oral exposure. A different effect can be obtained in some cases, when dsRNA is directly injected into the body of specimen toxic or supplied *via* ingestion (Powell et al., 2017). Therefore, toxicity tests should incorporate a dietary exposure to the test substance to ensure physiological exposure of specimen to dsRNA. A designed NTO study of sufficient length should allow for the elucidation of sublethal and lethal effects. Apical endpoints such as growth, development, reproduction/fecundity, and mortality are clearly linked to population level effects in NTOs and thus can be related back to protection goals.

Sub-lethal effects need to be duly considered, as gene silencing is expected in many cases not to induce acute lethal effects, but alteration of the physiology of target organisms may lead to a delayed effect and/or induce transient effects (e.g. Vargas et al., 2008; Kumar et al., 2012). In addition, studies of gene expression in the tested species where a matching sequence with the dsRNA has been observed in genomic analyses can ensure that the possible effect is indeed caused by silencing and not by environmental conditions during testing, which may affect gene expression in the target/non-target species.

*In planta* tests may also be necessary to ensure that safety for NTOs is demonstrated under environmental conditions that allow optimal gene expression in NTOs exposed to (near isogenic) control plants as well as test plants (Arpaia et al., 2017). Information regarding susceptibility of ‘control’ specimen in different conditions is then to be provided.

Another case in which *in planta* tests are applicable for ERA, is the study of pathogen resistance induced *via* RNAi, for instance, by modulating the expression of membrane surface proteins. This type of proteins cannot be tested in isolation, therefore *in planta* tests can be effectively used to assess impacts on NTOs (e.g. endophytic and mycorrhizal fungi) exposed directly or indirectly to dsRNA in GM plants.

Also in this area of ERA of RNAi plants, an additional source of available information in support to ERA is the availability of bioinformatics tools. However, due to the limited availability of genome sequencing for non-target organisms, and the restricted predictability of RNAi off-target genes in animals, bioinformatics cannot reliably determine the possibility of unintended silencing effects on those species but may represent a first screening, which may need further confirmation with the support of more traditional toxicity bioassays. Routinely screening the genomes of non-target organisms to identify genes that might be silenced, may not be practical due to the absence of sequence information on many NTOs. It may be possible to collect relevant information from the observation of off target silencing in the intended target species in order to estimate potential effects on NTOs, especially if they are taxonomically close to the target species.

## Stacked Events

In some cases, GM RNAi plants may be transformed to express more than one dsRNA, for example to silence a plant gene to confer a quality change as well as pest resistance. In these cases, the risk assessment should follow the EFSA guidance approach (EFSA, 2007) and carefully consider whether any interactions occur between the transgenes and/or the traits that may alter their expression or effects on targets and non-targets. For example, silencing a plant gene which has antifeedant activity may render the plant more susceptible to pests and thus affect pest plant relationship.

In addition, GM RNAi plants may contain transgenes conferring activity against the same or other pests and pathogens and providing other characteristics such as herbicide tolerance. For example maize MON 87411 expresses dsRNA targeting *DvSnf7* gene in *Diabrotica* spp., is also transformed to express a Cry3 toxin lethal to the same species. Other GM RNAi plants may contain transgenes stacked by hybridisation of lines with single and numbers of events such as other pest resistance genes, herbicide tolerance or other complimentary traits (e.g. maize MON87427×MON89034×MIR162×MON87411). In these cases the non RNAi GM events require the normal risk assessment procedures and so no changes in data requirements are envisaged for plants containing stacked RNAi and non-RNAi events for both ERA and food/feed safety evaluation. However, the risk assessments should carefully consider whether any interactions occur between the transgenes and/or traits, which may alter their expression or effects on target and non-target organisms. In the case of maize MON 87411 the expression of both the dsRNA and Cry3 toxin will result in different effects in the target organism. The acute toxicity of Cry3 may limit the exposure of *Diabrotica* individuals to dsRNA through feeding, whereas *Diabrotica* with some resistance to Cry 3 will feed for longer and receive higher doses of dsRNA. Studies of non-target organisms should consider the effects of both traits together where they are combined in single events.

In GM plants with events stacked by hybridisation it is also important to note that there can be segregation of events in subsequent generations. As advised in the EFSA Guidance note on stacks (EFSA, 2007), all potential novel combinations of events should be risk assessed as well as the stack and the single events.

## Discussion

Genetically modified plants expressing interfering RNAs represent a new generation of GM plants for which more applications for commercialization are expected in the near future. Some aspects of their physiology make them quite different from the “first generation” of currently marketed GM plants expressing a few phenotypic traits (mostly insect resistance *via* expression of Cry toxins or herbicide tolerance). For instance, RNAi plants for insect resistance exploit completely new mechanisms of action (e.g. targeting the endosomal sorting complexes required for insect cellular transport in the SmartStaxPro^®^ maize).

Therefore, in species with significant sequence similarity, underlying physiological mechanisms need to be considered in the context of the overall mechanism of action and previous history of use for products targeting some physiological functions in plant-dwelling organisms. Knowledge gaps (for example possible effects of the dsRNA in non-target organisms as well as off-target effects in the plant genome) need to be specifically tackled by applicants during risk assessment.

In their evaluation of Monsanto’s GM maize MON 87411 and the stack, MON 89034 x TC1507 x MON 87411 x DAS-59122-7 combined trait maize (SmartStax^®^ PRO) expressing a dsRNA targeting the Western Corn Rootworm *Snf7* gene, USEPA reviewed the extensive data set provided by the applicant on the effects of dsRNA in the context of its application in agriculture.

In considering potential human health risks from the dsRNA, USEPA’s risk assessment was based on the evidence provided, including a 28-day toxicology study on the dsRNA, supporting the findings from a USEPA Scientific Advisory Panel (USEPA, 2014; USEPA, 2016a). The report indicated that “no reliable evidence that exogenous dsRNAs are taken up from the gut” existed. The Panel concluded that the combination of RNAses and acids founds in the human digestive system ensure that all forms of RNAs expressed in plant material and consumed by humans are likely to be degraded.

When considering the ecological risk assessment of DvSnf7 RNA in the GM maize events, USEPA analyzed the battery of laboratory tests on non-target organisms including invertebrate predators, parasitoids, pollinators, soil biota, and aquatic and terrestrial vertebrate species. Data presented were considered a reasonable framework for future environmental assessments of pesticide products based on environmental dsRNA (USEPA, 2017). As a new mode of action was involved, the DvSnf7 RNA ecological risk assessment was also reviewed by a FIFRA Science Advisory Panel (SAP). Several aspects of the risk assessment approach, including exposure assumptions, environmental fate and non-target effects data, on toxicity and possible synergism with stacked *Bt* traits were considered. Furthermore, the risk assessment went on to state that “*in silico* evaluations are not considered to be predictive of adverse effects” (USEPA, 2017), and that such evaluations are currently only considered as supplemental information to provide additional evidence for risk determination.

Taken together, the data in support of the approval of GM maize events were considered adequate by EPA and they concluded that the application of RNAi-based mode of action for pest control in agriculture presents minimal hazard and risk to non-target organisms with protection goals (USEPA, 2016b; USEPA, 2017).

Likewise, Food Standard Australia and New Zealand applies a case-by-case approach to GM food safety assessment, which is considered sufficiently broad and flexible to address the safety of GM foods developed using gene silencing techniques such as RNAi technology (FSANZ, 2013).

The EFSA GDs are the technical support for applicants conducting risk assessment of GM plants according to the European legislative framework. The GDs indicate the general principles for conducting risk assessment, purposefully leaving room for selecting the necessary information for preparing dossiers case by case.

In this paper, we considered the main principles described in the current EFSA GDs for risk assessment of GM plants, to determine which areas of the existing risk assessment approaches for GM plants are appropriate or could be refined, and if complementary or alternative risk assessment strategies need to be developed for RNAi plants in the EU. We aimed at highlighting the rationale for defining specific biosafety data requirements for the risk assessment and risk management of RNAi plants and their derived products (i.e. food and feed).

The outcomes of our analyses, suggest that data requirements for the risk assessment of RNAi plants will be similar to other GM plants and therefore the risk assessment framework used so far for other GM plants is still valid. Likewise, the case by case approach depending on plant species, event and trait also applies for case specific post market monitoring. Guidelines for risk assessment cannot be “cookbooks” and some flexibility should be left for risk assessors to adapt and justify the details of their assessment. It is up to regulatory agencies to judge the validity of risk assessment approaches and support applicants in delivering estimates of risk at the highest safety standard, considering the severity and the likelihood of possible impacts on human, animal and environmental safety.

The approach outlined in this paper could provide support to future updates of the EFSA GDs, since there is not yet much experience, especially for environmental risk assessment, of RNAi plants. In particular, we wish to highlight a few characteristics of RNAi mechanisms, which need consideration during specific steps of the risk assessment (e.g. molecular characterization, NTO species selection).

### A Simplified Risk Assessment for Food and Feed Derived From RNAi Plants

Risk assessment of RNAi plants may need less data in some steps of the process. For instance, the molecular analysis and comparative compositional analyses between the GM plants and their comparators might show that no new proteins are produced and endogenous protein levels are unchanged. In this case, assessment of toxicity and allergenicity of new plant products are unnecessary. However according to the implementing regulation (EU 503/2013), 90-days feeding studies on rodents are currently mandatory regardless of the additional data available from chemical analyses and their interpretation.

### The Role of Bioinformatics

Bioinformatics may have an important role in supporting the risk assessment of RNAi plants, e.g. through opportune comparisons of the genomes (even if only partially known) of target and relevant non-target organisms which might be exposed to the interfering RNA in the field. In fact, a thoughtful design of dsRNA at the beginning of the process of development of new RNAi plants, can limit the possibility of non-target effects due to sequence similarity (USEPA, Scientific Advisory Panel, 2014). It is known that the taxonomic and genomic proximity of target and non-target species renders silencing effects more likely (Christiaens et al., 2018), however the extent of these effects may be variable between species in the same family (Haller et al., 2019). The limited availability of insect genomes in currently accessible databases further limits the predictive ability of bioinformatics, so that supporting data for an absence of non-target effects needs to be obtained through bioassays. However, results from bioinformatics analyses may contribute to build a weight of evidence on the safety assessment of RNAi plants.

### Specifically Tailored Bioassays

Experimental protocols for bioassays may need to be adapted in order to achieve the required sensitivity in detecting possible effects. While exposing test specimens in laboratory conditions to dsRNA *via* injection is a useful tool for elucidating silencing mechanisms, this pathway cannot be considered biologically relevant for estimating *in vivo* exposure of TOs and NTOs in nature; therefore ingestion of RNAi plant tissues or diet-incorporated dsRNA should be adopted for testing NTOs. Experimental conditions should ensure that the expression of the target gene is optimal in the specimen not exposed to dsRNA containing diet for the whole duration of the test. Enzymatic barriers in some species may degrade dsRNA, so the silencing effect in a given species might not occur in other taxa. Additional measurement endpoints (e.g. measurement of expression of the target gene) can increase confidence on the results of the bioassays. For instance, an analysis of silencing effects on the target gene (and possibly on a few essential off-target genes with sequence similarities) can clarify the physiological mechanisms determining phenotypical characters (i.e. mortality and sub-lethal effects).

Observations of the presence of dsRNA in different tissues and over the growth period of plants will be necessary to estimate exposure of target and some selected non-target organisms. In addition, data will be required on expression levels of dsRNA in TOs to enable the selection of significant pathways necessary to estimate exposure of their natural enemies to the dsRNA.

A special remark concerns the risk assessment of GM RNAi trees, some of which have already been authorized for commercialization outside the EU. Though it is not unique for RNAi-based events, the possible applications for commercial release of GM rootstocks will need a reflection on what kind of information will be needed regarding scions, and consequently on the fruits produced by such varieties. Current European regulation states that if part of a plant is GM then the whole plant is designated as GM. Consequently, a product of a GM plant, even if does not contain transgenic DNA, is classified as GM. In the European system, approval is given for a GM event. Once the event is approved it can be put into any genetic background through hybridization. For instance, if the same GM *Prunus* rootstock is used for plum, cherry, peach, apricot scions, then no new application is required.

Finally, reference is to be made to the recent developments of RNAi-based pesticide products for external application. While these products are subject to pesticide regulation, risk assessment requirements and protocols are likely to be derived from experiences with GM RNAi plants. However, there are some unique aspects, which will have to be considered for externally applied RNAi products, such as issues of plant uptake, effect of chemical modifications, carriers or formulations on dsRNA stability, the effects of stabilizing measures on the exposure and impacts of non-target species (Taning et al., 2020).

## Conclusions

A working group of the COST Action iPlanta discussed the main aspects, relevance and applicability of the principles of the existing EFSA guidelines to environmental and food/feed risk assessment for RNAi plants. The authors consider that the current science-based regulatory process in Europe is still applicable to RNAi plants; nevertheless, the assessment process should permit some flexibility for risk assessors to adapt and justify the case-by-case assessment of their RNAi plants.

We highlight the following considerations linked to the peculiarity of RNAi GM plants that could be also considered for further updates of the existing EFSA Guidance Documents on risk assessment for GMOs:

- The data related to newly expressed proteins, protein equivalence and the codon optimization are irrelevant for the inserted DNA as long as no part is translated to protein. Consequently, the food and feed safety assessment could be simplified with regard to novel proteins and their potential allergenicity if no novel proteins are produced. Intended as well as unintended effects triggered by siRNA in the plant can be detected by compositional analyses and, where applicable, nutritional evaluation of food and feed derived from RNAi plants. Potential siRNA effects in humans and farm animals through dietary dsRNA/siRNA are highly unlikely because of rapid degradation in the GI tract and several barriers to cellular uptake in mammals. The data on levels of dsRNA expression over time, in different plant tissues and related to environmental conditions during the experiments are necessary for estimating the possible exposure to the dsRNA in plants or derived food or feed for consumers and non-target organisms in the receiving environment;
- Bioinformatics can offer a good support to risk assessment, especially when designing dsRNA sequences specific to the target gene and minimizing the potential for off-target binding sites. Due to the limitations in genome sequences for NTOs, for ERA bioinformatics analyses should be complemented with specifically developed bioassays and measurement of gene suppression.

## Author Contributions

This article is the result of a meeting of the working group “Specific biosafety issues associated with RNAi” of the iPlanta COST Action, held in Rome in October 2019 and organized by SA, AD-P, and BM. All co-authors gave a lecture at the meeting and contributed to the writing of the article based on the internal notes of the meeting prepared by SA and AD-P. All authors contributed to the article and approved the submitted version.

## Funding

The present paper is an output of the COST Action iPlanta CA 15223, funded by the European Commission. OC is a recipient of a postdoctoral fellowship of the Research Foundation—Flanders (FWO). The Institute of Biological, Environmental and Rural Sciences (IBERS) receives strategic funding from the Biotechnology and Biological Sciences Research Council (BBSRC) *via* Grant BB/CSP1730/1.

## Conflict of Interest

KG is employed by the company Bayer. The remaining authors declare that the research was conducted in the absence of any commercial or financial relationships that could be construed as a potential conflict of interest.
